# Emotional Salience and Interpersonal Problems in Depressed Older Adults With Personality Pathology

**DOI:** 10.1093/geroni/igaa057.1479

**Published:** 2020-12-16

**Authors:** Avner Aronov, Helene Geramian, Jennifer Ho, Wing Jin Mak, Ira Yenko, Hyunyoung Ellen Park, Dimitry Francois, Richard Zweig

**Affiliations:** 1 Yeshiva University, Ferkauf Graduate School of Psychology, Brooklyn, New York, United States; 2 Yeshiva University Ferkauf Graduate School of Psychology, The Bronx, New York, United States; 3 Ferkauf Graduate School of Psychology, Yeshiva University, The Bronx, New York, United States; 4 Yeshiva University Ferkauf Graduate School of Psychology, Mountain View, California, United States; 5 Yeshiva University Ferkauf Graduate School of Psychology, Bronx, New York, United States; 6 Weill Cornell Medicine, White Plains, New York, United States

## Abstract

Research demonstrates reciprocal relationships between personality and depression as well as the important role interpersonal conflicts play, but rarely explores these risk factors in older adults. This study aimed to examine relationships of personality traits, processes, and the impact of emotional involvement and distress during an interpersonal conflict on depression in older adults. The study also investigated whether emotional involvement or interpersonal distress moderate the relationship between personality pathology and depression. Depressed middle and older adult inpatients (N=37; mean age=65.73, SD=7.81; 56.8% female; 86.5% White/Non-Hispanic) completed self-reports and interview-based assessments regarding personality traits (NEO-FFI Neuroticism, Agreeableness), interpersonal problems (IIP-25), and depression (GDS). Narrative responses regarding an interpersonal conflict were obtained and rated for contamination themes as well as emotional involvement and distress. Overall, findings indicated that living with others predicted higher depression (p= .046) and was related to higher neuroticism and interpersonal problems. Personality traits (Neuroticism) (r= .485, p= .001) and processes (Interpersonal problems-trend) (β= .307, p= .058), as well as higher levels of emotional distress (r= .486, p= .001) and involvement (r= .475, p= .001) in an interpersonal conflict were also tied to depression in bivariate but not multivariate analyses. The moderating effects of emotional involvement or distress on the relationship between personality and depression were not supported. Depressed older inpatients who live with others appear at higher risk of depression. Personality traits and processes may be more distal risk factors for depression. Findings are discussed in relation to stress generation as well as clinical implications targeting emotional regulation.

